# The sleep quality and influencing factors among midwives in China: a cross-sectional study

**DOI:** 10.3389/fpubh.2025.1581508

**Published:** 2025-07-07

**Authors:** Dongning He, Jinling Zhang, Mingxin Zhu, Yangxue Meng, Na Li, Guoyu Wang, Biru Luo

**Affiliations:** ^1^Department of Nursing, West China Second University Hospital, Sichuan University/West China School of Nursing, Sichuan University, Chengdu, Sichuan, China; ^2^Key Laboratory of Birth Defects and Related Diseases of Women and Children (Sichuan University), Ministry of Education, Chengdu, Sichuan, China; ^3^Department of Obstetric Nursing, West China Second University Hospital, Sichuan University/West China School of Nursing, Sichuan University, Chengdu, Sichuan, China

**Keywords:** sleep, sleep quality, sleep deprivation, midwives, occupational health

## Abstract

**Objective:**

This study aimed to assess sleep quality and identify influencing factors among midwives in mainland China, providing a reference for obstetric managers to develop strategies that enhance sleep health among midwives.

**Methods:**

The study was conducted from January to December 2023 across 566 hospitals in mainland China. A total of 1,948 midwives participated. A self-designed questionnaire was used to collect socio-demographic information and participants' perceived factors influencing their sleep quality. Sleep quality was assessed using the Pittsburgh Sleep Quality Index (PSQI), and regression analysis was performed to identify the factors influencing sleep quality.

**Results:**

The median Pittsburgh Sleep Quality Index score was 9, with 71.9% (*n* = 1,400) of midwives reporting poor sleep quality (cutoff score >7). Additionally, 12.4% of midwives required medication to assist with sleep. Statistically significant differences (*P* < 0.05) in scores were observed across variables such as years of experience as a midwife, health status, hospital type, work mode, ability to leave work on time, and sleep assistance situation. Regression analysis indicated that health status, hospital type, work mode, ability to leave work on time, the need for sleep assistance, and perceived work pressure were significant factors influencing sleep quality among midwives.

**Conclusion:**

The overall sleep quality of Chinese midwives is poor, influenced by both work-related and individual factors. Obstetric managers should allocate clinical staff and resources efficiently. This can be achieved by adjusting shift rotations, reducing overtime, and implementing other measures to create an environment that supports better sleep quality among midwives, enhancing their work performance and ensuring maternal and neonatal safety.

## 1 Introduction

Sleep is a biological necessity and a positive process that restores energy and regulates emotions, which is an important component of health (1). However, due to increasing work demands and changes in lifestyle, sleep problems have become a common issue globally and have attracted increasing attention. Research indicated that the prevalence of sleep-related issues ranged from 14.5 to 56%, with variations among different countries (2–5). Sleep quality generally refers to subjective perceptions of sleep onset latency, total wakefulness, sleep efficiency, and the number of awakenings (6), and it is considered a significant indicator of overall health (7). Research reported that short-term poor sleep quality can lead to noticeable fatigue (8), reduced concentration, and impaired cognitive function (9). In the long term, poor sleep quality is strongly associated with an increased risk of metabolic diseases such as obesity and diabetes (10), as well as cardiovascular diseases (11). Furthermore, poor sleep quality is closely linked to mental health conditions (12), including anxiety and depression. Additionally, prolonged poor sleep quality weakens immune system functioning, reducing the body's resistance to infections and illnesses (13). These findings highlight the critical importance of paying attention to sleep quality, especially for individuals in high-stress professions (14). Healthcare workers are especially susceptible to the impacts of sleep.

The nature of clinical work necessitates rotation and night shifts (15), requiring healthcare workers to stay alert during work hours and respond promptly to emergencies. Sleep quality serves as an important foundation for supporting such high-intensity work, directly influencing job performance and clinical decision-making (16, 17). Insufficient sleep and poor sleep quality can lead to issues such as lack of concentration, slower reactions, and emotional instability among healthcare workers. Research has indicated that these issues may increase their risk of occupational injuries, such as needlestick injuries, sharp object injuries, and motor vehicle accidents due to drowsy driving (18, 19). Additionally, these problems could also increase the risk of medical errors and pose potential threats to patient safety (20, 21). Research has shown that 39.2%−61% of healthcare workers experience sleep problems (22–24). Nurses and midwives are particularly affected due to the physical demands of their work, frequent night shifts, and direct interactions with patients or pregnant women, resulting in a higher prevalence of sleep-related issues within this group compared to doctors (24). Midwives, as professionals specializing in vaginal delivery support (25), have responsibilities that differ from nurses. Besides providing high-quality obstetric care for women during the perinatal period (26), midwives are required to independently manage labor progress, assist in delivery, and, when necessary, decide on and perform procedures such as episiotomies and wound suturing. Moreover, obstetric emergencies such as placental abruption, amniotic fluid embolism, and postpartum hemorrhage require midwives to quickly form a rescue team with obstetricians (27). These tasks significantly increase the demands on midwives' professional skills and decision-making abilities (28). Therefore, good sleep quality not only helps midwives better alleviate fatigue and restore physical energy but is also crucial for maintaining their cognitive function and professional capacity.

Although the work of midwives is characterized by high intensity and specialty, current research often combines nurses with midwives and neglects the unique professional characteristics and the special nature of obstetric work. Furthermore, previous research on the occupational health of midwives has paid insufficient attention to sleep quality. This study is conceptually informed by the Effort-Recovery Model (29), which posits that work-related effort and stress require adequate recovery periods to prevent negative health outcomes. When recovery is incomplete, due to factors such as irregular work schedules, overtime, or persistent work-related stressors, individuals may experience impaired physiological and psychological functioning, including poor sleep quality. This framework guided our examination of the relationships between workload, work stress, and sleep quality among midwives. To enrich this research, we conducted a national cross-sectional study to assess the sleep quality and influencing factors of Chinese midwives. This study aims to provide a reference for obstetric managers in implementing specialized sleep support measures, improving the work environment for midwives, and ultimately enhancing maternal and neonatal care quality.

## 2 Methods

### 2.1 Setting

This cross-sectional study was conducted from January 2023 to December 2023 across hospitals of different levels in mainland China to investigate sleep quality and its influencing factors among midwives. The midwives included in the study were recruited from public hospitals. Hospitals were categorized into general hospitals and specialized hospitals. In this study, specialized hospitals were defined as facilities that provide health services exclusively for women and children. In the work mode of midwives, “Day shift only” refers to working 8–12 h during the daytime with 1–2 days off per week. “Day shift with regular night shift” refers to a fixed rotation cycle of 1–2 days of day shifts followed by one night shift, and then 1–3 days off. “Day shift with irregular night shift” refers to a schedule with regular day shifts but irregular night shifts, with both shift patterns and rest days unfixed.

### 2.2 Participants

This study used convenience sampling to recruit participants from different levels of public general and specialized hospitals within the coverage areas of 10 standardized midwifery training bases of the China Maternal and Child Health Association. These bases are evenly distributed across the eastern, central, and western regions of China, covering most provinces, autonomous regions, and directly administered cities in mainland China. The inclusion criteria for the midwives in this study were: (1) regular employees of the maternity ward; (2) able to work independently in their job duties (without requiring continuous supervision or guidance from a clinical mentor); and (3) having normal listening, reading, and writing skills. The exclusion criteria were: (1) being involved in other sleep-related research projects; and (2) having a clear diagnosis of psychiatric disorders (e.g., depression, anxiety disorders).

### 2.3 Measurements

The socio-demographic information of participants was collected through a self-designed questionnaire, which included gender, age, years of experience as a midwife, education level, professional title, health status, marital status, fertility status, hospital level and type, work mode, ability to leave work on time, and sleep assistance situations. In addition, the questionnaire included questions about factors that midwives might consider as influencing their sleep quality, such as sleep environment, emotional status, work pressure, on-call work requirements, additional work during rest time, commuting time, and responsibilities for elder care. In this study, “additional work during rest time” refers to work-related tasks that midwives are required to complete during their scheduled rest periods, such as attending meetings, completing documentation, or participating in various training sessions. The Pittsburgh Sleep Quality Index (PSQI) was used to assess the sleep quality of the midwives. It was developed by Buysse et al. (30) in 1989 and is a self-report questionnaire that evaluates various aspects of sleep. It consists of 19 items across seven dimensions, including sleep quality, sleep latency, sleep duration, sleep efficiency, sleep disturbances, use of sleeping medication, and daytime dysfunction. Each dimension is scored from 0 to 3, and the total score ranges from 0 to 21, with a higher PSQI score indicating poorer sleep quality. This study adopted a cutoff score of 7 to classify sleep quality; a score ≤7 was considered good sleep quality, and a score >7 indicated poor sleep quality. While the original PSQI validation study recommended a cutoff of >5, with a reported sensitivity of 89.6% and specificity of 86.5% in general populations (30), validation studies in Chinese populations have demonstrated that a cutoff of >7 provides substantially higher diagnostic accuracy, with a sensitivity of 98.3% and specificity of 90.2% (31). This higher threshold has been shown to more effectively differentiate between good and poor sleep quality in Chinese populations and has been widely adopted in recent Chinese studies on healthcare workers (32, 33).

### 2.4 Data collection

We used Survey Star (a specialized online platform for creating surveys) to design an online questionnaire. The questionnaire link was distributed through the official workgroup of the China Maternal and Child Health Association to the managers of standardized midwifery training bases. These managers were then asked to communicate with the maternity ward managers in public hospitals within the coverage areas of their respective bases, via email or WeChat (a popular social application in China), to collect the email addresses of those who were willing to participate in the survey at their hospitals. Four investigators received training from the main researchers before distributing the questionnaires, and each was responsible for liaising with two or three midwifery training bases. The training was considered successful if the investigators were able to clearly explain the instructions for completing each section of the questionnaire. After completing the training, the investigators sent the questionnaire link and inclusion and exclusion criteria of participants to the email addresses of the maternity ward managers, asking them to forward the questionnaire to the midwives who met the criteria. To facilitate communication, the investigators established WeChat groups for each base. If maternity ward managers or participating midwives had any questions about the questionnaire, they could contact the investigators via email, phone, or WeChat. Before the questionnaire was presented, a page appeared explaining the objective of the study and requesting informed consent from participants, who were required to provide an electronic signature. Only after completing the informed consent, the participants could continue filling out the questionnaire. Every participant had the right to decide whether to participate in the research and could withdraw at any time. After each questionnaire was collected, two independent investigators reviewed the completed questionnaires to identify logical inconsistencies or missing responses, questionnaires with more than 20% missing data were discarded. Finally, we received a total of 2,017 questionnaires from 566 hospitals of different levels and types. The response rate was 84.7%.

### 2.5 Data statistics

IBM SPSS 27.0 was used to analyze data. The results showed that the continuous variables did not follow a normal distribution. Therefore, the PSQI dimension scores and total scores were expressed as medians (interquartile ranges, IQR), and categorical data were presented as frequencies (*n*) and percentages (%). Univariate analysis was conducted using Pearson's chi-square test to identify potential factors influencing sleep quality among the midwives. Binary multivariable logistic regression analysis was performed to estimate the odds ratios (OR) and 95% confidence intervals (CI). Variables significantly associated with the outcome in the univariate analysis (*P* < 0.05) were simultaneously included in the binary multivariable logistic regression model to determine their associations with midwives' sleep quality. A two-tailed *P*-value of < 0.05 was considered statistically significant.

### 2.6 Ethics approval

This study was approved by the Ethics Committee of West China Second University Hospital, Sichuan University, with ethics approval number 2022 (322). Informed consent was obtained from all participants. All collected data were fully anonymized before analysis. No identifying information was collected, and individual participants could not be re-identified from the dataset. This approach ensured compliance with data privacy and confidentiality standards.

## 3 Results

### 3.1 The sociodemographic characteristics of participants

A total of 1,948 midwives were finally included in the study. Of the participants, 66.8% (*n* = 1,302) were from public tertiary A hospitals, and 65.2% (*n* = 1,271) were from general hospitals. The main work mode of the participants was the day shift with regular night shifts (69.3%). A total of 640 (32.9%) participants needed non-drug or drug assistance to sleep. Other socio-demographic characteristics are shown in Table 1.

**Table 1:** Sociodemographic characteristics and sleep quality of midwives (*N* = 1,948).

**Variables**	***N* (%)**	**Sleep quality**		***P*-value**
		**Good sleep** ***n*** = **548 (%)**	**Poor sleep** ***n*** = **1,400 (%)**	
**Gender**				**0.237**
Male	14 (0.7)	6 (1.1)	8 (0.6)	
Female	1,934 (99.3)	542 (98.9)	1,392 (99.4)	
**Age, year**				**0.102**
< 25	73 (3.8)	29 (5.3)	44 (3.1)	
25–34	1,117 (57.3)	301 (54.9)	816 (58.3)	
35–44	616 (31.6)	174 (31.8)	442 (31.6)	
≥45	142 (7.3)	44 (8.0)	98 (7.0)	
**Years of experience as a midwife, year**				**0.030**
< 5	422 (21.7)	143 (26.1)	279 (19.9)	
5–9	665 (34.1)	177 (32.3)	488 (34.9)	
10–14	462 (23.7)	120 (21.9)	342 (24.4)	
≥15	399 (20.5)	108 (19.7)	291 (20.8)	
**Educational level**				**0.335**
Junior college and below	334 (17.2)	100 (18.2)	234 (16.7)	
Undergraduate	1,582 (81.2)	436 (79.6)	1,146 (81.9)	
Master and above	32 (1.6)	12 (2.2)	20 (1.4)	
**Professional title**				**0.116**
Junior midwife	194 (10.0)	65 (11.9)	129 (9.2)	
Senior midwife	797 (40.9)	213 (38.9)	584 (41.7)	
Supervising midwife	794 (40.8)	216 (39.4)	578 (41.3)	
Associate chief midwife and above	163 (8.3)	54 (9.8)	109 (7.8)	
**Health status**				<**0.001**
Excellent	332 (17.0)	164 (29.9)	168 (12.0)	
Good	703 (36.1)	255 (46.5)	448 (32.0)	
Fair	827 (42.5)	123 (22.5)	704 (50.3)	
Poor	86 (4.4)	6 (1.1)	80 (5.7)	
**Marital status**				**0.428**
Single	375 (19.3)	112 (20.4)	263 (18.8)	
Married	1,530 (78.5)	427 (77.9)	1,103 (78.8)	
Divorced	43 (2.2)	9 (1.7)	34 (2.4)	
**Fertility status**				**0.502**
No children	547 (28.1)	156 (28.5)	391 (27.9)	
One child	1,031 (52.9)	297 (54.2)	734 (52.4)	
Two or more children	370 (19.0)	95 (17.3)	275 (19.7)	
**Hospital level**				**0.250**
Public tertiary A hospital	1,302 (66.8)	356 (65.0)	946 (67.6)	
Public tertiary B hospital	270 (13.9)	88 (16.0)	182 (13.0)	
Public secondary A hospital	322 (16.5)	86 (15.7)	236 (16.8)	
Public secondary B hospital and below	54 (2.8)	18 (3.3)	36 (2.6)	
**Hospital type**				<**0.001**
General hospital	1,271 (65.2)	392 (71.5)	879 (62.8)	
Specialized hospital	677 (34.8)	156 (28.5)	521 (37.2)	
**Work mode**				<**0.001**
Day shift only	333 (17.1)	132 (24.1)	201 (14.4)	
Day shift with regular night shift	1,349 (69.3)	374 (68.2)	975 (69.6)	
Day shift with irregular night shift	266 (13.6)	42 (7.7)	224 (16.0)	
**Ability to leave work on time**				<**0.001**
Yes	957 (49.1)	331 (60.4)	626 (44.7)	
No	991 (50.9)	217 (39.6)	774 (55.3)	
**Sleep assistance situations**				<**0.001**
No assistance	1,308 (67.1)	478 (87.2)	830 (59.3)	
Non-drug assisted sleep	399 (20.5)	63 (11.5)	336 (24.0)	
Drug-assisted sleep	241 (12.4)	7 (1.3)	234 (16.7)	

### 3.2 Midwives perceived factors affecting sleep quality

The perceived factors affecting sleep quality among midwives are shown in Table 2. A total of 67.6% of midwives believed that sleep environment could influence sleep quality. One thousand five hundred and fifty-two (79.7%) thought that emotional status was an influencing factor. Regarding work-related factors, more than 80% of the participants believed that work pressure could affect sleep quality, and more than half of midwives considered on-call work requirements as a factor. However, 54.9% of midwives did not think additional work during rest time influenced sleep quality. Additionally, 1,493 participants did not consider commuting time to be an influencing factor and 1,536 participants did not believe that responsibilities for elder care affected sleep quality.

**Table 2:** Sleep quality of midwives and perceived factors affecting sleep (*N* = 1,948).

**Variables**	***N* (%)**	**Sleep Quality**		***P*-value**
		**Good sleep** ***n*** = **548 (%)**	**Poor sleep** ***n*** = **1,400 (%)**	
**Sleep environment**				**0.179**
Yes	1,317 (67.6)	358 (65.3)	959 (68.5)	
No	631 (32.4)	190 (34.7)	441 (31.5)	
**Emotional status**				<**0.001**
Yes	1,552 (79.7)	406 (74.1)	1,146 (81.8)	
No	396 (20.3)	142 (25.9)	254 (18.2)	
**Work pressure**				<**0.001**
Yes	1,639 (84.1)	414 (75.5)	1,225 (87.5)	
No	309 (15.9)	134 (24.5)	175 (12.5)	
**On-call work requirements**				<**0.001**
Yes	1,023 (52.5)	237 (43.2)	786 (56.1)	
No	925 (47.5)	311 (56.8)	614 (43.9)	
**Additional work during rest time**				<**0.001**
Yes	879 (45.1)	204 (37.2)	675 (48.2)	
No	1,069 (54.9)	344 (62.8)	725 (51.8)	
**Commuting time**				<**0.001**
Yes	455 (23.4)	86 (15.7)	369 (26.3)	
No	1,493 (76.6)	462 (84.3)	1,031 (73.7)	
**Responsibilities for elder care**				<**0.001**
Yes	412 (21.2)	83 (15.1)	329 (23.5)	
No	1,536 (78.9)	465 (84.9)	1,071 (76.5)	

### 3.3 The PSQI score and sleep quality level of participants

The participants' PSQI dimension scores and sleep quality levels are shown in Table 3. The total PSQI score was 9 (6, 12). Among the participants, 28.1% (*n* = 548) reported good sleep quality, while 71.9% (*n* = 1,400) were classified as having poor sleep quality. The subjective sleep quality scores indicated that at least 25% of midwives rated their sleep quality as poor or very poor. The sleep latency scores showed that 50% or more of the midwives took over 31 min or more to fall asleep. The sleep duration scores revealed that at least half of the midwives slept for <6 h per night. The sleep efficiency scores indicated that fewer than 75% of midwives had a sleep efficiency of ≥85%. The sleep disturbance scores showed that at least a quarter of the midwives experienced nighttime awakenings, nightmares, or breathing difficulties once or twice a week or more. The use of sleep medication scores demonstrated that at least 75% of participants never used medication to assist with sleep. The daytime dysfunction scores indicated that at least 75% of midwives reported varying degrees of daytime dysfunction, while at least 25% of midwives experienced daily dysfunction such as difficulty concentrating, fatigue, and emotional disturbances.

**Table 3:** The PSQI score and sleep quality level of midwives (*N* = 1,948).

**Score of PSQI**	**Median**	**IQR**
Sleep quality	1	1, 2
Sleep latency	2	1, 2
Sleep duration	2	1, 2
Sleep efficiency	0	0, 1
Sleep disturbances	1	1, 2
Use of sleeping medication	0	0, 0
Daytime dysfunction	2	1, 3
Total	9	6, 12
**Sleep quality**	***N***	**%**
Good (≤7)	548	28.1
Poor (>7)	1,400	71.9

### 3.4 The sleep quality comparison of midwives with different characteristics

The comparison of sleep quality among participants with different socio-demographic characteristics and their perceived factors influencing sleep quality are shown in Tables 1, 2, respectively. The results showed that midwives with years of experience as a midwife, health status, those working in hospitals of different levels and types, and those with different work modes, ability to leave work on time, and sleep assistance needs, had statistically significant differences in sleep quality (all *P* < 0.05). Except for the sleep environment, midwives who perceived emotional status, work pressure, on-call work requirements, additional work during rest time, commuting time, and responsibilities for elder care as influencing factors, their sleep quality had statistically significant differences in sleep quality (all *P* < 0.05).

### 3.5 The influencing factors of sleep quality among midwives

The variables that had statistically significant differences in sleep quality among midwives, as determined by Pearson's chi-square test, were entered into the binary multivariable logistic regression model. The regression results are shown in Table 4. The results indicated that years of experience as a midwife did not have statistically significant differences in the regression model (*P* > 0.05). Health status was an influencing factor for sleep quality among midwives. Compared with excellent health status, midwives with a lower level of health status had a higher risk of poor sleep quality. Midwives who worked in specialized hospitals had a lower likelihood of good sleep quality compared with those who worked in general hospitals (*P* = 0.014, OR = 0.741, 95% CI = 0.583, 0.941). Midwives whose work mode was day shifts only were more likely to have good sleep quality compared with those whose work mode required regular or irregular night shifts. Work mode was a factor influencing sleep quality. The ability to leave work on time was a factor influencing sleep quality. Midwives who were able to leave work on time had better sleep quality than those who were unable to (*P* = 0.003, OR = 1.417, 95% CI = 1.123, 1.787). Midwives who needed non-drug (OR = 0.453, 95% CI = 0.332, 0.616) or drug (OR = 0.081, 95% CI = 0.037, 0.174) assistance to help with sleep had a lower likelihood of having good quality sleep. Among the perceived influencing factors, those who thought work pressure could influence their sleep quality had a higher risk of poor sleep quality (*P* = 0.008, OR = 0.667, 95% CI = 0.494, 0.900). Other factors they perceived did not enter the regression model.

**Table 4:** Logistic regression analysis of factors influencing the sleep quality of midwives.

**Variables**	**β**	**SE**	**Wald**	***P***	**OR (95% CI)**
**Years of experience as a midwife (Ref:**<**5)**					
5–9	−0.275	0.150	3.377	0.066	0.759 (0.566, 1.018)
10–14	−0.229	0.166	1.908	0.167	0.795 (0.574, 1.101)
≥15	−0.264	0.181	2.116	0.146	0.768 (0.538, 1.096)
**Health status (Ref: Excellent)**					
Good	−0.436	0.144	9.187	0.002	0.647 (0.488, 0.857)
Fair	−1.335	0.157	72.302	<0.001	0.263 (0.193, 0.358)
Poor	−1.735	0.456	14.471	<0.001	0.176 (0.072, 0.431)
Hospital type (Ref: General hospital)	−0.300	0.122	6.062	0.014	0.741 (0.583, 0.941)
**Work mode (Ref: Day shift only)**					
Day shift with regular night shift	−0.370	0.152	5.878	0.015	0.691 (0.513, 0.932)
Day shift with irregular night shift	−0.723	0.229	9.993	0.002	0.485 (0.310, 0.760)
Ability to leave work on time (Ref: No)	0.348	0.118	8.642	0.003	1.417 (1.123, 1.787)
**Sleep assistance situations (Ref: No assistance)**					
Non-drug assisted sleep	−0.793	0.157	25.350	<0.001	0.453 (0.332, 0.616)
Drug-assisted sleep	−2.519	0.393	40.997	<0.001	0.081 (0.037, 0.174)
Emotional status (Ref: No)	−0.143	0.140	1.046	0.306	0.867 (0.659, 1.140)
Work pressure (Ref: No)	−0.405	0.153	7.023	0.008	0.667 (0.494, 0.900)
On-call work requirements (Ref: No)	−0.177	0.121	2.141	0.143	0.837 (0.660, 1.062)
Additional work during rest time (Ref: No)	0.046	0.126	0.132	0.716	1.047 (0.817, 1.341)
Commuting time (Ref: No)	−0.245	0.152	2.598	0.107	0.783 (0.582, 1.054)
Responsibilities for elder care (Ref: No)	−0.213	0.153	1.947	0.163	0.808 (0.599, 1.090)

## 4 Discussion

In this study, we investigated the current sleep quality of midwives in China. Our findings revealed that 71.9% of midwives had poor sleep quality, with a PSQI score of 9 (interquartile range 6–12), indicating that at least half of the midwives may be experiencing severe sleep disorders (31), which is higher than the result of another sleep quality study on Chinese midwives (60.12%) that used the same measurement and cutoff (32). Although there was a difference between the studies, the results still highlighted the prevalence of sleep issues among midwives. In high-pressure medical environments, adequate rest and good sleep quality for healthcare workers are essential for maintaining effective clinical operations. Recognizing this, we further explored the factors associated with midwives' sleep quality. We identified significant work environment factors, including hospital type, work mode, and the ability to leave work on time. Additionally, individual factors such as health status, methods of sleep assistance, and recognition of the impact of work pressure on sleep quality were also contributing factors.

### 4.1 The work environment influencing factors of sleep quality among midwives

Regarding the work background of midwives, we found that those working in specialized hospitals had poorer sleep quality. This difference is likely associated with the delivery volumes and workloads in different types of hospitals in China. Specifically, the obstetrics department in general hospitals tends to have lighter workloads than other departments. Compared to general hospitals, specialized hospitals typically have more obstetrics wards and beds, which may be associated with a higher volume of deliveries and, consequently, an increased workload for midwives. Additionally, the childbirth rate in China has continued to decline in recent years (34), which has resulted in the closure of obstetrics wards in some secondary general hospitals and specialized hospitals (35). As a result, an increasing number of pregnant women are choosing tertiary specialized hospitals for childbirth, which in turn may contribute to an increased workload for midwives in these institutions. However, the higher workload may be associated with midwives working longer hours and having more frequent shift rotations, which may elevate both their physical and psychological stress (36) and may contribute to poorer sleep duration and quality. Although the results indicated that midwives in specialized hospitals had poorer sleep quality, we cannot change the type of hospital they work in. However, this highlights the need to pay greater attention to the sleep quality of midwives in specialized hospitals. Obstetric managers should consider addressing the impact of hospital type by ensuring sufficient human resource allocation, reasonable working hours, and an effective shift rotation system. The study indicated that compared with midwives on day shifts, those on night shifts are at greater risk of poor sleep quality, especially those with irregular night shifts. This is consistent with previous research on shift workers (37–39). The sleep-wake schedule of day-shift midwives matches the circadian rhythm (40), which may help them achieve more continuous sleep at night. This may be the primary reason why their sleep quality is better than that of night-shift midwives. Midwives with regular night shifts demonstrated better sleep quality compared to those with irregular night shifts. Although working night shifts inevitably disrupts normal routines and circadian rhythms, midwives with regular night shifts may find it easier to establish an adaptive sleep schedule and maintain a consistent sleep pattern (41). However, midwives with irregular night shifts may adapt to a new routine, only to have it disrupted again. Lim et al. (42) found that frequently changing shifts prevent the circadian clock from adjusting, leading to a mismatch between hormonal secretion and the actual sleep-wake cycle, which may contribute to midwives with irregular night shifts having poorer sleep quality. This result suggests that when night shifts for midwives are unavoidable, obstetric departments should prioritize implementing regular night shift rotations to improve their sleep quality, and the specific frequency of night shifts and the rest periods following these shifts should be carefully evaluated and adjusted in consideration of actual human resource availability. Additionally, we found that midwives who were able to leave work on time had better sleep quality. This might be because leaving work on time may help them maintain a stable routine and better organize their personal time, thereby contributing to a healthier work-life balance and supporting both physical and psychological recovery (43). Research has shown that working overtime often increases job-related stress and can even lead to career burnout (44), and may significantly impact sleep quality (45). For midwives working night shifts, failing to leave work on time could disrupt their established post-night-shift sleep routine (46), which may result in fragmented sleep and may negatively impact overall sleep quality (47). This issue requires particular attention. Therefore, obstetric managers should minimize midwives' overtime whenever possible, especially during night shifts, to ensure they have adequate recovery time. If unavoidable circumstances require midwives to work overtime, adequate rest should be provided as compensation. Managers could offer a certain degree of flexibility in shift rotations or extend rest periods after overtime to reduce the negative impact on sleep quality (48). These findings align well with the Effort-Recovery Model (29), which emphasizes that adequate recovery periods are essential for preventing the negative health consequences of work-related effort and stress. Our results demonstrate that when midwives lack sufficient recovery opportunities—due to factors such as high workload, irregular shifts, and insufficient rest—their sleep quality may be compromised.

### 4.2 The individual influencing factors of sleep quality among midwives

The study showed that health status is significantly associated with sleep quality, with midwives in better health generally experiencing better sleep quality, consistent with previous research (49). Similarly, Cheng et al. (50) reported that individuals with poor health may experience more sleep disorders, which may contribute to a decline in sleep quality. Therefore, when considering shift rotations, managers should take midwives' health status into account and allocate shifts rationally (49). This could help ensure the operation of clinical work while potentially contributing to better sleep quality among midwives, thereby promoting their physical and psychological well being. Compared to midwives who do not require sleep assistance, those using non-drug methods or medications to aid sleep were less likely to experience good sleep quality, especially those who relied on medication. This phenomenon may be associated with various factors. Firstly, non-drug methods such as music therapy can be somewhat effective in improving sleep quality; however, their efficacy is relatively insufficient for severe sleep disorders (51). Although medication can promote rapid sleep onset, it may disrupt the structure of sleep by reducing deep sleep and rapid eye movement phases (52), which may impact the recovery of midwives' bodies and minds. Additionally, the side effects of sleep medicine, such as drowsiness or dizziness (53), may further impair midwives' work performance and sleep quality, with potential implications for maternal and neonatal health. To address this issue, we suggest that managers closely attend to the sleep assistance needs of midwives and implement supportive measures. For instance, establishing a quiet restroom equipped with aromatherapy and soft lighting may help midwives fall asleep naturally. Additionally, organizing sleep health courses may enhance midwives' understanding of sleep assistance methods. Furthermore, managers should assess workforce allocation and resource distribution to ensure a balance between midwives' work demands and their need for adequate rest. Our research revealed that midwives who perceived work stress as influencing their sleep quality tended to experience poorer sleep quality. This association may be related to heightened stress sensitivity and the development of sleep-related anxiety (54). These midwives might excessively focus on their sleep status and tend to associate fluctuations in sleep quality with work stress. Such heightened attention could contribute to the development of sleep-related anxiety, which may subsequently disrupt the process of falling asleep and may negatively impact overall sleep quality. Additionally, these midwives may lack effective strategies for regulating and coping with work stress. The research showed that individuals who are unable to cope with work stress are more likely to experience insomnia and other sleep problems (55).

Therefore, we suggest that obstetric managers address midwives' work stress and sleep. This could potentially be achieved by organizing regular stress management training programs to improve midwives' stress regulation and sleep management skills. Additionally, managers should establish robust psychological support mechanisms, offering personalized counseling services or group support activities tailored to midwives' needs. Although obstetric managers cannot directly intervene in the individual factors affecting midwives' sleep quality, they can make adjustments to the work environment to reduce the impact of these factors on their sleep.

### 4.3 Limitation

This study was a cross-sectional survey, which identified the factors influencing midwives' sleep quality. However, it cannot determine how these factors directly affect sleep quality. The PSQI, a self-report tool, was used to assess midwives' sleep quality. This method may introduce data bias, as participants might not accurately reflect their true sleep quality due to social desirability bias or memory recall bias. Future studies could combine objective methods, such as using wearable devices like sleep rings, to more accurately measure sleep quality. Additionally, we did not record the number or proportion of midwives who declined to participate. Since the survey invitations were distributed through base managers to maternity ward managers, it was difficult to track refusals accurately. Furthermore, selection bias may have occurred if ward managers selectively invited midwives whom they believed were more likely to participate. As a result, midwives from private hospitals, rural healthcare settings, or those in non-standard employment arrangements may have been underrepresented in our sample. These factors may affect the representativeness of the sample. Finally, all participants in this study were from public hospitals in China, and the sample did not include other types of hospitals. This means the results may not be applicable to other hospital settings, particularly midwives working in private or rural hospitals.

## 5 Conclusion

This study indicated that the overall sleep quality of Chinese midwives is poor, with both work-related and individual factors influencing their sleep quality. Obstetric managers should ensure the smooth operation of clinical services while making efforts to understand midwives' health status, sleep assistance methods, and sleep perceptions. They should also allocate clinical staff and resources rationally. By adjusting shift rotations and reducing overtime, obstetric managers can provide a foundational environment that supports improvements in midwives' sleep quality, thereby enhancing their work performance and ensuring maternal and neonatal safety in the future.

## Data Availability

The original contributions presented in the study are included in the article/supplementary material, further inquiries can be directed to the corresponding authors.
